# Supplementary material for: Esterase Activity of Serum Albumin Studied by 1H NMR Spectroscopy and Molecular Modelling
Source: Int J Mol Sci. 2021 Sep 30;22(19):10593. doi: 10.3390/ijms221910593 (PMC8508922; doi:10.3390/ijms221910593)
Supplement: Supplementary file 1 [file ijms-22-10593-s001.zip › ijms-1388654-supplementary.pdf]

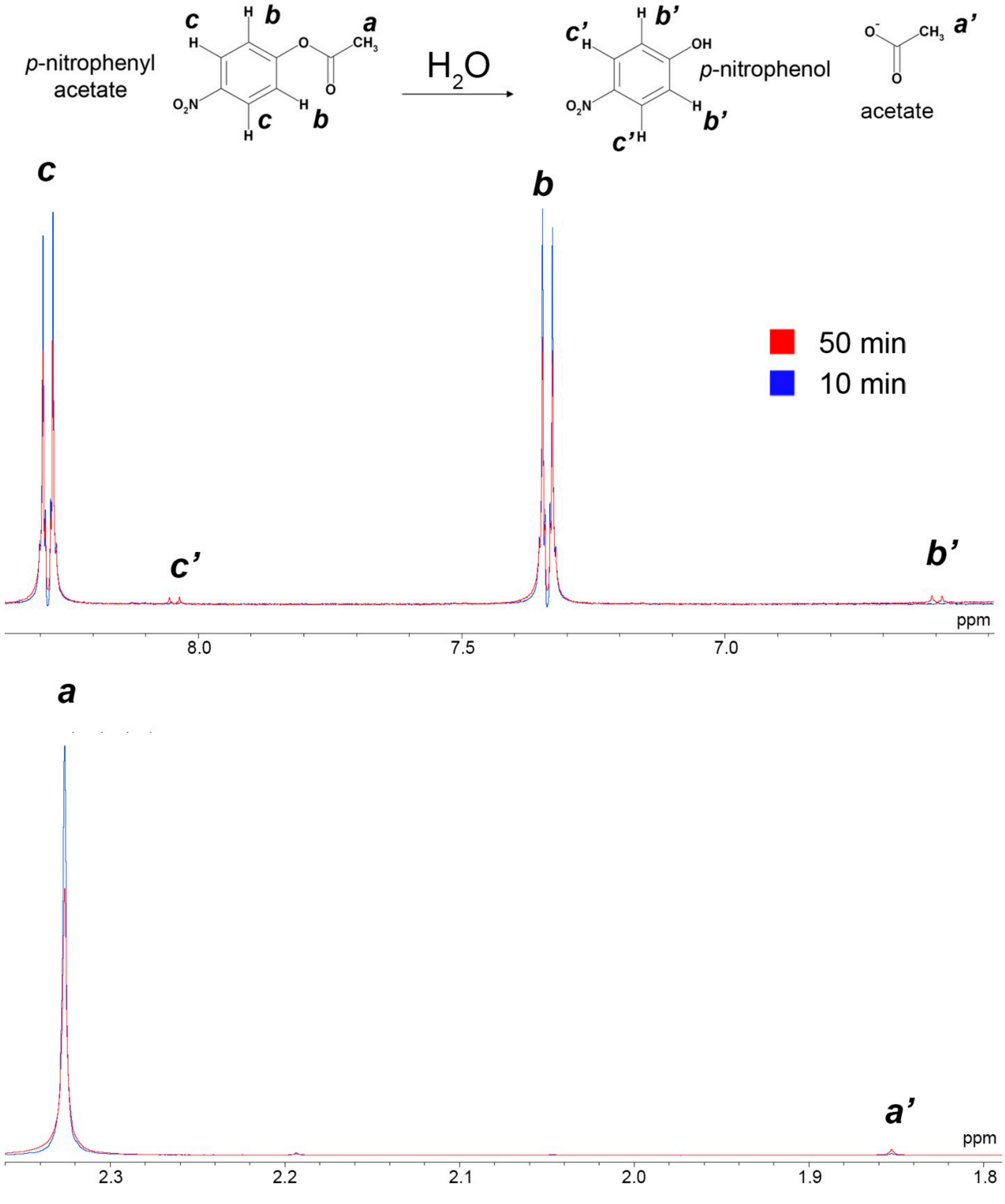

Figure S1. Kinetics of spontaneous hydrolysis of *p*-nitrophenyl acetate (NPA) according to ^{1}H NMR data: aromatic part of the spectrum (top) and aliphatic part of the spectrum (bottom).

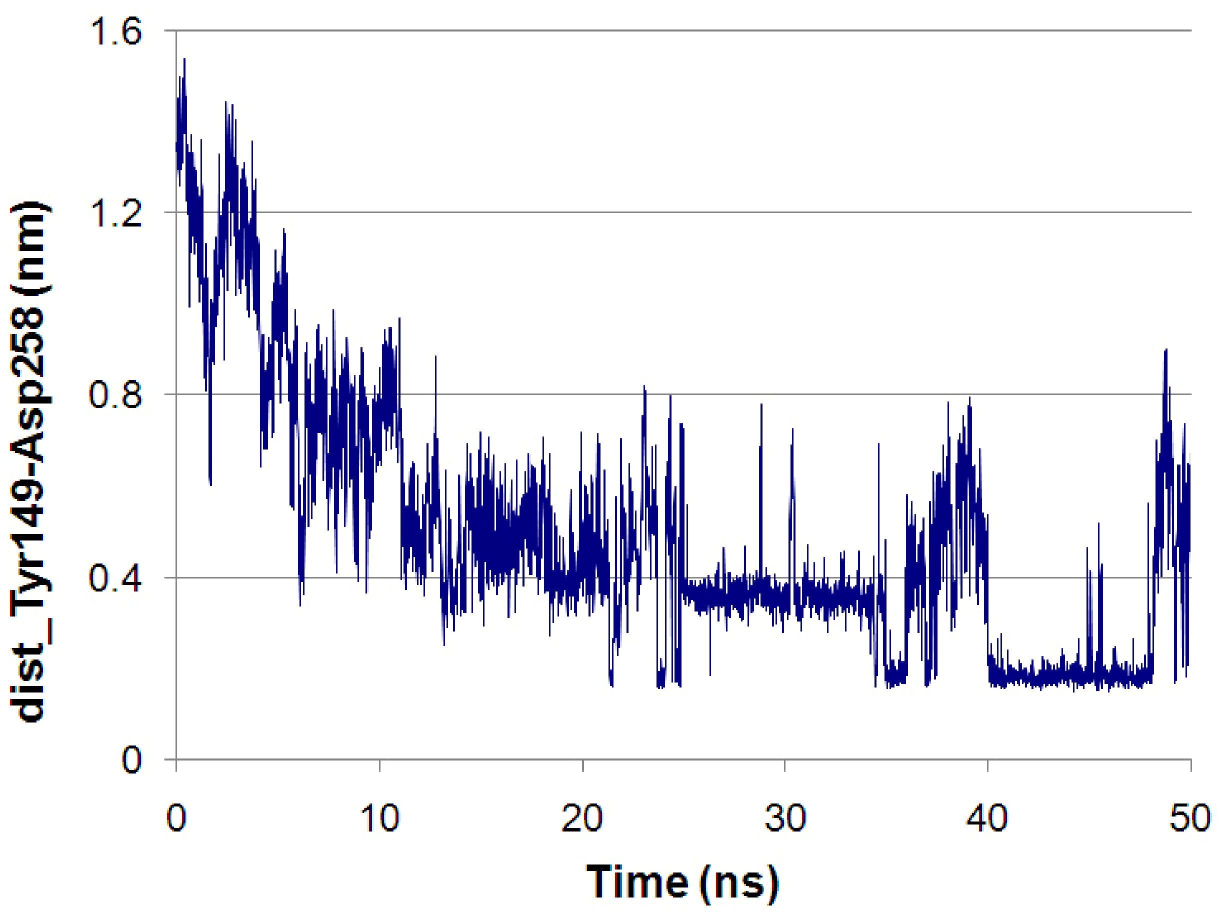

Figure S2. Time change of the distance (dist_Tyr149-Asp258) between the hydroxyl oxygen atom of catalytic tyrosine Tyr149 and atom Oδ1 of Asp258 of bovine serum albumin.
